# Selectively Disrupted Functional Connectivity Networks in Type 2 Diabetes Mellitus

**DOI:** 10.3389/fnagi.2015.00233

**Published:** 2015-12-11

**Authors:** Yaojing Chen, Zhen Liu, Junying Zhang, Guihua Tian, Linzi Li, Sisi Zhang, Xin Li, Kewei Chen, Zhanjun Zhang

**Affiliations:** ^1^State Key Laboratory of Cognitive Neuroscience and Learning, IDG/McGovern Institute for Brain Research, Beijing Normal University, Beijing, China; ^2^BABRI Centre, Beijing Normal University, Beijing, China; ^3^Dongzhimen Hospital, Beijing University of Chinese Medicine, Beijing, China; ^4^School of Life Sciences, Fudan University, Shanghai, China; ^5^Banner Alzheimer’s Institute, Phoenix, AZ, USA

**Keywords:** type 2 diabetes mellitus, Alzheimer’s disease, resting state network, functional magnetic resonance imaging, functional connectivity

## Abstract

**Background:**

The high prevalence of type 2 diabetes mellitus (T2DM) in individuals over 65 years old and cognitive deficits caused by T2DM have attracted broad attention. The pathophysiological mechanism of T2DM-induced cognitive impairments, however, remains poorly understood. Previous studies have suggested that the cognitive impairments can be attributed not only to local functional and structural abnormalities but also to specific brain networks. Thus, our aim is to investigate the changes of global networks selectively affected by T2DM.

**Methods:**

A resting state functional network analysis was conducted to investigate the intrinsic functional connectivity in 37 patients with diabetes and 40 healthy controls who were recruited from local communities in Beijing, China.

**Results:**

We found that patients with T2DM exhibited cognitive function declines and functional connectivity disruptions within the default mode network, left frontal parietal network, and sensorimotor network. More importantly, the fasting glucose level was correlated with abnormal functional connectivity.

**Conclusion:**

These findings could help to understand the neural mechanisms of cognitive impairments in T2DM and provide potential neuroimaging biomarkers that may be used for early diagnosis and intervention in cognitive decline.

## Introduction

The dramatically increased worldwide prevalence of diabetes, especially in individuals over 65 years old (Polonsky, 2012), has attracted extensive attention. In China, diabetes has also become a major public health problem with the prevalence increasing with age and weight (Yang et al., 2010). Type 2 diabetes mellitus (T2DM), which comprises the majority of diabetes cases, can damage many domains of cognitive function involving memory, executive function, visuo-spatial ability, and attention (Kodl and Seaquist, 2008). Nevertheless, the pathophysiological mechanism of cognitive deficits induced by T2DM remains poorly understood.

Several previous studies have found that T2DM was associated with atrophy of the gray matter (Bryan et al., 2014) and microstructural abnormalities in the white matter (Zhang et al., 2014). Moreover, they suggested that cognitive impairments caused by T2DM could be attributed to those changes in local structure (Zhang et al., 2014). Similarly, middle-aged subjects with type 1 diabetes mellitus (T1DM) had injured white matter in the posterior corona radiata and the optic radiation and the reduced fractional anisotropy of these white matter tracts correlated with poorer cognitive performances (Kodl et al., 2008). Whether the cognitive decline is a direct consequence of these local abnormalities has not yet been confirmed. Based on this notion, some investigators have explained the cognitive impairments from the perspective of the white matter topological network and found that alterations in global network properties were related to the slowing of information-processing speed in T2DM patients (Reijmer et al., 2013).

Resting state functional magnetic resonance imaging (MRI) provides a promising and non-invasive neuroimaging technique to measure spontaneous or intrinsic brain activity (Biswal et al., 1995). Recently, when measured by blood oxygen level dependent (BOLD) functional MRI (fMRI) in the resting awake brain, spontaneous fluctuation signals could be delineated as the human neural functional architecture, which includes the default mode network (DMN), somatomotor network, right and left frontal–parietal network (FPN), and primary visual network (Damoiseaux et al., 2006). Changes in the resting state networks (RSNs) include a decrease activity in the DMN (Damoiseaux et al., 2008) and an increase in functional density in the somatosensory and subcortical networks (Tomasi and Volkow, 2012) with aging. At the same time, there is a close relationship between T2DM and neurodegenerative disorders in elderly, especially the Alzheimer’s disease (AD) (Arvanitakis et al., 2004), and it has been well documented that AD seem to be directly associated with individual networks, including the DMN, salience network (SN), and executive control networks (ECN) (Dai et al., 2014). The network dysfunctions could also predict AD progression that the sensorimotor and attention networks were attacked at early stage and then extended to the key DMN and SN in AD patients (Wang et al., 2015). In addition, our previous study of silent lacunar infarcts in the basal ganglia region showed that disrupted intrinsic connectivity within/between the DMN and SN correlated with the cognitive performance of patients (Chen et al., 2014c). These studies suggested that injury to specific RSNs is caused by neurological diseases. Since T2DM could damage cognitive performance and increase the onset risk of neurodegenerative disorders, such as AD, we wondered the relationship between T2DM and some intrinsic functional networks. Previous resting state studies in T2DM patients have focused on changes in spontaneous brain activity (Cui et al., 2014) and the connectivity of the hippocampus (Zhou et al., 2010) and posterior cingulate cortex (Chen et al., 2014d). Noticeably, recent studies have demonstrated that insulin resistance, a common symptom of T2DM, correlated with functional connectivity in the middle temporal gyrus (MTG) (Xia et al., 2015) and the posterior part of DMN (Cui et al., 2015). Besides, one study in T1DM found that patients showed decreased connectivity in some RSNs involving attention and working memory, but only in patients with microangiopathy (Van Duinkerken et al., 2012). The study also demonstrated a positive relationship between information-processing speed, and general cognitive ability and degree of connectivity (Van Duinkerken et al., 2012). To the best of our knowledge, however, the relationship between the alteration of the functional RSNs and cognition deficits in T2DM patients has not been determined.

In the present study, we enrolled 37 patients with T2DM and 40 age-, gender-, and education-matched healthy controls to conduct clinical assessments, a battery of neuropsychological tests and resting state fMRI. In this study, group independent component analyses (ICA) were applied to investigate global RSNs in T2DM patients and healthy controls. Our aim is to find the unique functional networks affected by diabetes and hypothesized changed connectivity in DMN. We expect that our study could provide insight into the pathogenic mechanisms of T2DM-related cognitive impairment.

## Materials and Methods

### Participants

The participants in this study were all from the Beijing Aging Brain Rejuvenation Initiative (BABRI), which is an ongoing, longitudinal study investigating aging, and cognitive impairment in urban elderly people in Beijing, China. In this cross-sectional study, all of the participants were selected according to the following criteria: (1) no <6 years of education; (2) scores ≥24 on the Mini-Mental-Status Examination-Chinese version (MMSE); (3) no history of coronary disease, nephritis, tumors, gastrointestinal disease, or psychiatric illness; and (4) able to meet the physical demands of the imaging procedure. A total of 77 participants met these criteria, including 37 patients with diabetes and 40 healthy controls. T2DM was diagnosed using established criteria based on medical histories, medication use, or fasting plasma glucose (FPG) levels of ≥7 mmol/l (Mayfield, 1998). All subjects provided a medical history and underwent a physical examination, during which height, weight, and body mass index (BMI) were recorded. The FPG, glycosylated hemoglobin (HbA1C), and cholesterol levels were measured with standard laboratory tests. Of the 37 T2DM patients, 6 patients were undergoing treatment with insulin, and 31 patients controlled fasting blood glucose using oral hypoglycemic agents (23 with glucosidase inhibitors, 5 with glurenorm, and 3 with biguanides). All participants self-reported to have never experienced severe hypoglycemia and had a low frequency of minor hypoglycemia. This study was conducted according to the principles of the Declaration of Helsinki and was approved by the institutional review board (IRB) at the Imaging Center for Brain Research at Beijing Normal University. All participants gave written informed consent for this study.

Vascular risk factors are indeed the established risk factors of diabetes. In our study, all participants completed the personal information questionnaire, which included smoking habits and medical records of hypertension. In particular, we categorized smoking into non-, past, and current smokers of <10 and ≥10 cigarettes per day. Of the 37 patients with T2DM, 32 participants were self-reported non-smokers, 4 past, and 1 current smoked <10 cigarettes per day. Of the 40 controls, 37 participants self-reported to have never smoked and 3 were past smokers. Regarding the hypertension, five T2DM patients and two controls had it.

### Neuropsychological Testing

All participants were subjected to a battery of neuropsychological tests that assessed their general mental status and other cognitive domains, such as memory, attention, spatial processing, executive function, and language abilities. As mentioned previously, their general mental status was assessed with the MMSE (Ming-Yuan, 1993), and patients with scores <24 were excluded because subjects with such low scores were considered to have possible dementia. The comprehensive neuropsychological battery is comprised of the following five cognition domains (the tests used to assess each domain are in parentheses): (1) memory [the Auditory Verbal Learning Test (AVLT) (Schmidt, 1996), the Rey–Osterrieth Complex Figure test (ROCF) (recall) (Rey, 1941) and the Digit Span test (Gong, 1992)]; (2) attention [the Trail-Making Test (TMT) A (Reitan, 1958), the Symbol Digit Modalities Test (SDMT) (Sheridan et al., 2006) and the Stroop Color and Word Test (SCWT) B (Golden, 1978)]; (3) visuo-spatial ability [ROCF-copy (Rey, 1941) and the Clock-Drawing Test (CDT) (Rouleau et al., 1992)]; (4) language [the Category Verbal Fluency Test (CVFT) and the Boston Naming Test (BNT) (Efgh and Weintraub, 1983)]; and (5) executive function [the TMT-B (Reitan, 1958) and the SCWT-C].

### Data Acquisition

Magnetic resonance imaging data were acquired using a SIEMENS TRIO 3-T scanner in the Imaging Center for Brain Research, Beijing Normal University. Participants were in a supine position with their head snugly fixed by straps and foam pads to minimize head movement. Resting state data were collected using a gradient echo EPI sequence (TE = 30 ms, TR = 2000 ms, flip angle = 90°, 33 slices, slice thickness = 4 mm, in-plane matrix = 64 × 64, field of view = 256 mm × 256 mm). During the single-run resting acquisition, the subjects were instructed to remain awake, relax with their eyes closed, and remain as motionless as possible. The resting acquisition lasted for 8 min, and 240 image volumes were obtained. Fluid-attenuated inversion recovery (FLAIR) and T1-weighted images were also acquired to measure the vascular brain lesions. The participants’ scans were separately reviewed by two experienced neurologists.

### Data Processing and Analysis

#### Preprocessing

For each participant, the first 10 volumes were discarded to allow the participants to adapt to the magnetic field. Functional data were preprocessed using SPM and DPARSF,^1^ and the processing included slice timing, within-subject interscan realignment to correct for possible movement, spatial normalization to a standard brain template in the Montreal Neurological Institute coordinate space, resampling to 3 mm × 3 mm × 3 mm, and smoothing with an 8-mm full-width half-maximum Gaussian kernel.

### Independent Component Analysis

We entered preprocessed resting fMRI data into an ICA using the group ICA toolbox (GIFT version 2.0e).^2^ The number of components (maps and corresponding time courses) estimated for each subject was set to 25. For each of the two groups, there are three main stages to group ICA: (i) principal component analysis was performed for each subject for data reduction, (ii) application of the ICA algorithm, and (iii) back reconstruction for each individual subject. After back reconstruction, the mean spatial maps of each group were converted to *z*-scores for display. The best-fit components for the binary FPN, DMN, sensorimotor network (SMN), medial visual network (MVN), and SN were identified by visual inspection. For each network, between-group comparisons were carried out using two-sample *t*-tests performed in SPM 8 (*q* < 0.05, FDR corrected).

For further analysis, region-of-interest (ROI) analysis was performed on any significant clusters resulting from the voxel-wise group comparisons. For each cluster with each network, the connectivity values were extracted by averaging the intensities over all voxels within the cluster from each participant’s component spatial map.

### Statistical Analysis

Independent two-sample *t*-tests were used to assess the between-group differences in age and years of education. The chi-square test was used to compare the gender ratios of the groups. For the neuropsychological assessment and biochemical indicator, an analysis of covariance (ANCOVA) was used to test for between-group differences (age, gender, and education were included as covariates). Pearson correlation analyses were performed to explore the relationship between the FPG level and the connectivity of the clusters with significant inter-group differences after controlling for the influences of age, gender, and education. All statistical analyses were performed using SPSS version 22 for Windows.

## Results

### Demographic and Clinical Characteristics and Neuropsychological Results

According to FLAIR and T1-weighted images, all participants had not white matter lesions. Of the 37 patients with T2DM, 4 participants had lacunar infarcts, 3 in the putamen, and 1 in the caudate nucleus. Of the 40 controls, 3 participants had lacunar infarcts, 2 in the putamen, and 1 in the thalamus. Although they have small ischemic lesion on gray matter, they have no symptoms. The groups did not differ significantly in age, gender, or years of education, vascular brain lesions, smoking habits, or medical history of hypertension. The neuropsychological and biochemical measures were analyzed with ANCOVA after adjusting for age, gender, and education. Compared with the control group, the patients with T2DM performed significantly poorer on the working memory tasks (Backward digit span, *p* = 0.02 and Digit span, *p* = 0.04), visuo-spatial processing task (ROCF-Copy, *p* = 0.01), and executive function task (SCWT C-B Time, *p* < 0.001). The patients with T2DM had significantly higher HbA_1_c, FPG, and BMI, triglyceride (TG), and low density lipoprotein (LDL) levels than the healthy controls (Table 1).

**Table 1:** **Demographic and neuropsychological test results**.

	Type 2 diabetes (*n* = 37)	Healthy controls (*n* = 40)	T/F-value (χ^2^)	*p*-Value
Age	64.27 ± 6.61	64.35 ± 5.72	−0.06	0.96
Male/female	17/20	19/21	0.02	1.00^a^
Education	11.55 ± 3.06	11.40 ± 2.49	0.24	0.81
Lacunar infarcts (%)	4 (10.8%)	3 (7.5%)	0.26	0.71^a^
Hypertension (%)	5 (13.5%)	2 (5%)	1.69	0.25^a^
Smoking				
Non/past/current	32/4/1	37/3/0	1.39	0.49^a^
General mental status				
MMSE	27.11 ± 2.49	27.48 ± 1.72	0.66	0.42
Memory function				
AVLT-delay recall	4.35 ± 3.00	4.55 ± 1.91	0.14	0.71
AVLT-T	25.97 ± 9.26	26.38 ± 7.69	0.06	0.81
ROCF-delay recall	12.19 ± 6.53	11.60 ± 4.68	0.17	0.68
Backward digit span	3.84 ± 1.34	4.60 ± 1.37	6.02	0.02
Digit span	11.11 ± 2.22	12.41 ± 3.35	4.46	0.04
Spatial processing				
ROCF-copy	32.32 ± 3.26	34.00 ± 2.11	7.39	0.01
CDT	24.11 ± 3.59	25.18 ± 5.17	1.01	0.32
Language				
CVFT	42.57 ± 11.63	43.28 ± 9.30	0.08	0.78
BNT	23.41 ± 3.32	23.40 ± 3.65	0.00	0.97
Attention				
SDMT	31.84 ± 11.98	34.10 ± 12.00	1.06	0.31
TMT-A time(s)	66.00 ± 35.02	57.78 ± 20.83	1.94	0.17
Executive function				
SCWT C-B time	43.76 ± 22.46	32.28 ± 14.45	8.80	<0.001
TMT-B time(s)	186.03 ± 83.89	167.48 ± 67.00	1.51	0.22
Biochemical indicator				
BMI	25.36 ± 2.40	23.63 ± 2.45	2.73	0.04
HbA_1c_ (%)	7.03 ± 0.68	5.50 ± 0.37	9.67	<0.001
HbA_1c_ (mmol/mol)	53.35 ± 7.53	36.65 ± 4.13	11.65	<0.001
FPG	7.34 ± 2.91	4.90 ± 0.53	6.18	<0.001
TC	4.74 ± 1.08	5.14 ± 0.78	2.86	0.10
TG	2.62 ± 3.51	1.43 ± 0.95	3.88	0.05
HDL	1.29 ± 0.54	1.36 ± 0.27	0.53	0.47
LDL	2.79 ± 0.98	3.22 ± 0.70	4.37	0.04

*Values are means ± SD or number of participants. The comparison of neuropsychological scores and biochemical indicator between the two groups was performed with an analysis of covariance*.

*^a^The p-value for gender, hypertension, and smoking ratio were obtained using a Chi-square test*.

*MMSE, Mini-Mental Status Examination; AVLT, Auditory Verbal Learning Test; ROCF, Rey–Osterrieth Complex Figure test; CDT, Clock-Drawing Test; CVFT, Category Verbal Fluency Test; BNT, Boston Naming Test; SDMT, Symbol Digit Modalities Test; SCWT, Stroop Color and Word Test; TMT, Trail-Making Test; HbA_1C_, glycosylated hemoglobin; FPG, fasting plasma glucose; TC, total cholesterol; TG, triglyceride; HDL, high density lipoprotein; LDL, low density lipoprotein; BMI, body mass index*.

### Altered RSNs in Patients with T2DM

**Figure 1:**
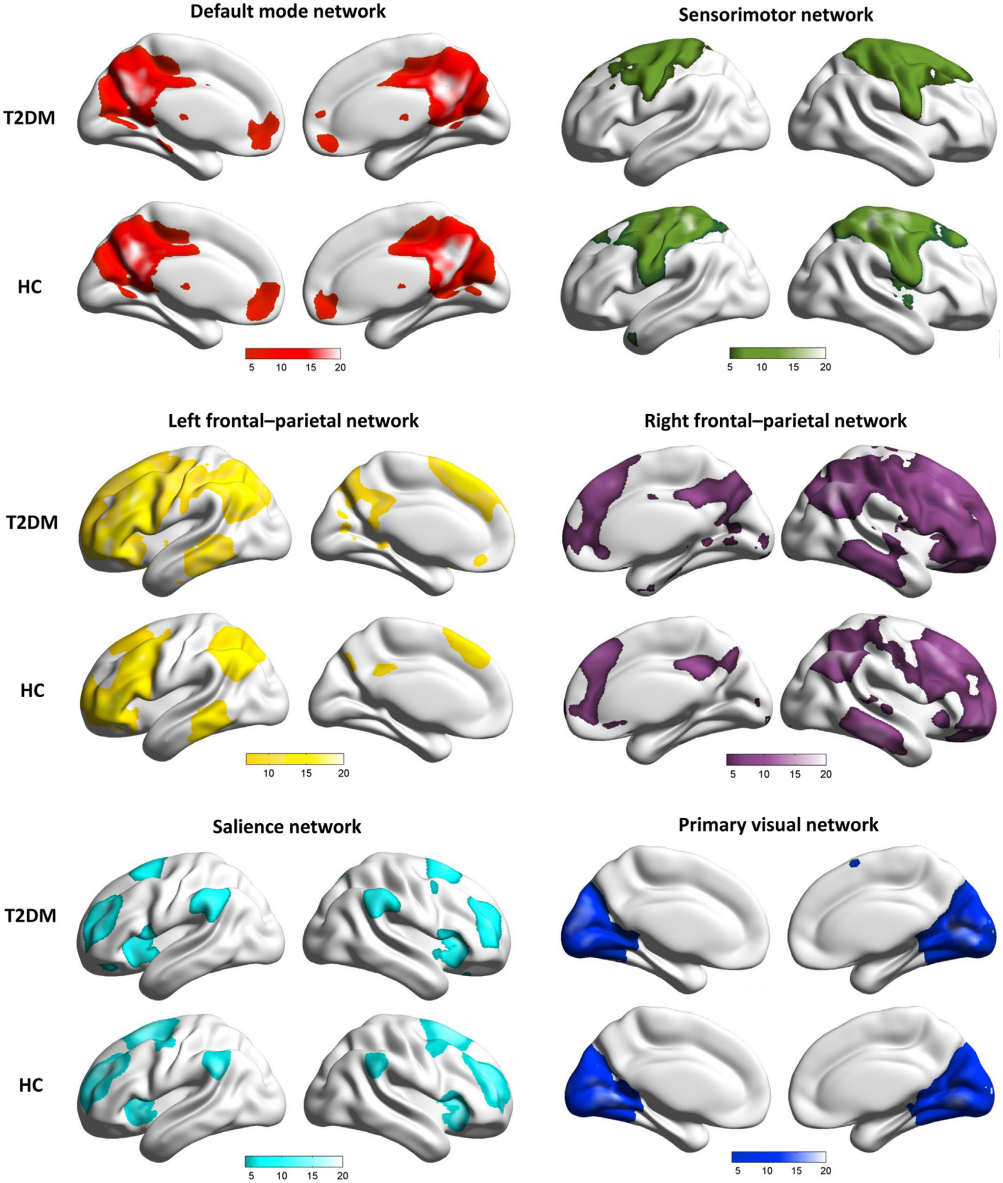
**Spatial maps of the six RSNs of patients with T2DM and normal controls**.

From the results of the group ICA, six functional brain networks of interest were identified: the SN, DMN, left FPN (LFPN), right FPN (RFPN), MVN, and SMN (Figure 1). The spatial maps described the anatomical regions involved for each group.

To quantify the observed changes within the brain networks, we investigated the effects of T2DM on the functional architecture of the brain by comparing the connectivity of the six RSNs between T2DM patients and controls.

**Figure 2:**
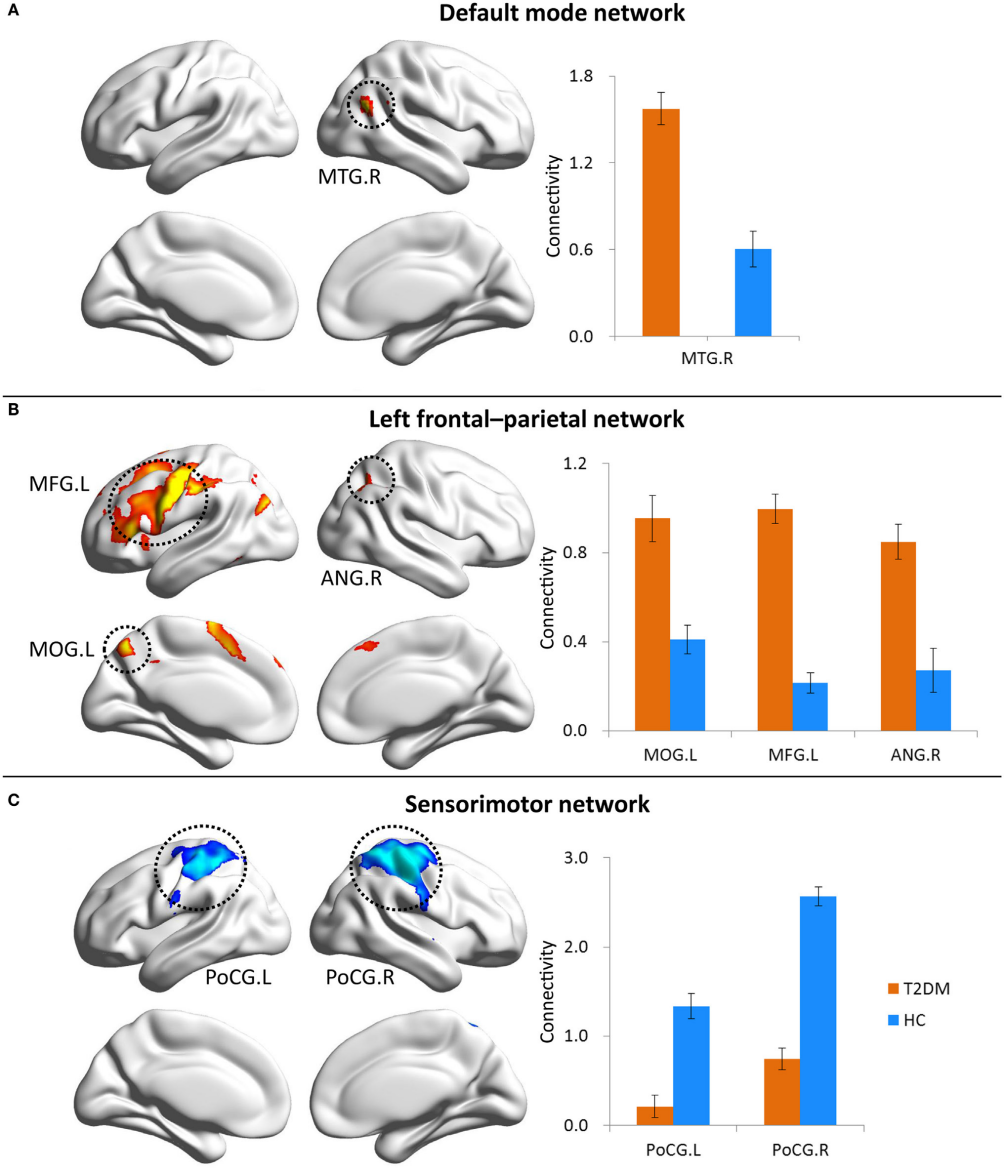
**Contrasts of RSNs between normal controls and patients with T2DM**. Brain maps represent voxel-wise group differences (FDR corrected, *q* < 0.05) for default mode network **(A)**, left frontal–parietal network **(B)**, and sensorimotor network **(C)**. Bar graphs show ROI analysis on the significant regions from voxel-wise comparisons. Error bars denote SEM.

In the DMN and LFPN, T2DM patients demonstrated higher connectivity relative to healthy controls (Figure 2). The significantly increased connectivity was located in the right MTG (MTG·R) within the DMN and the left middle occipital gyrus (MOG·L), left middle frontal gyrus (MFG·L), and right angular gyrus (ANG·R) within the LFPN (FDR corrected, *q* < 0.05). After correcting for multiple comparisons, the connectivity was diminished in the bilateral postcentral gyrus (PoCG) within the SMN in T2DM patients compared with controls. The average connectivity value from each region was extracted in patients and control subjects.

### Correlations Between FPG, Connectivity, and Cognitive Function

**Figure 3:**
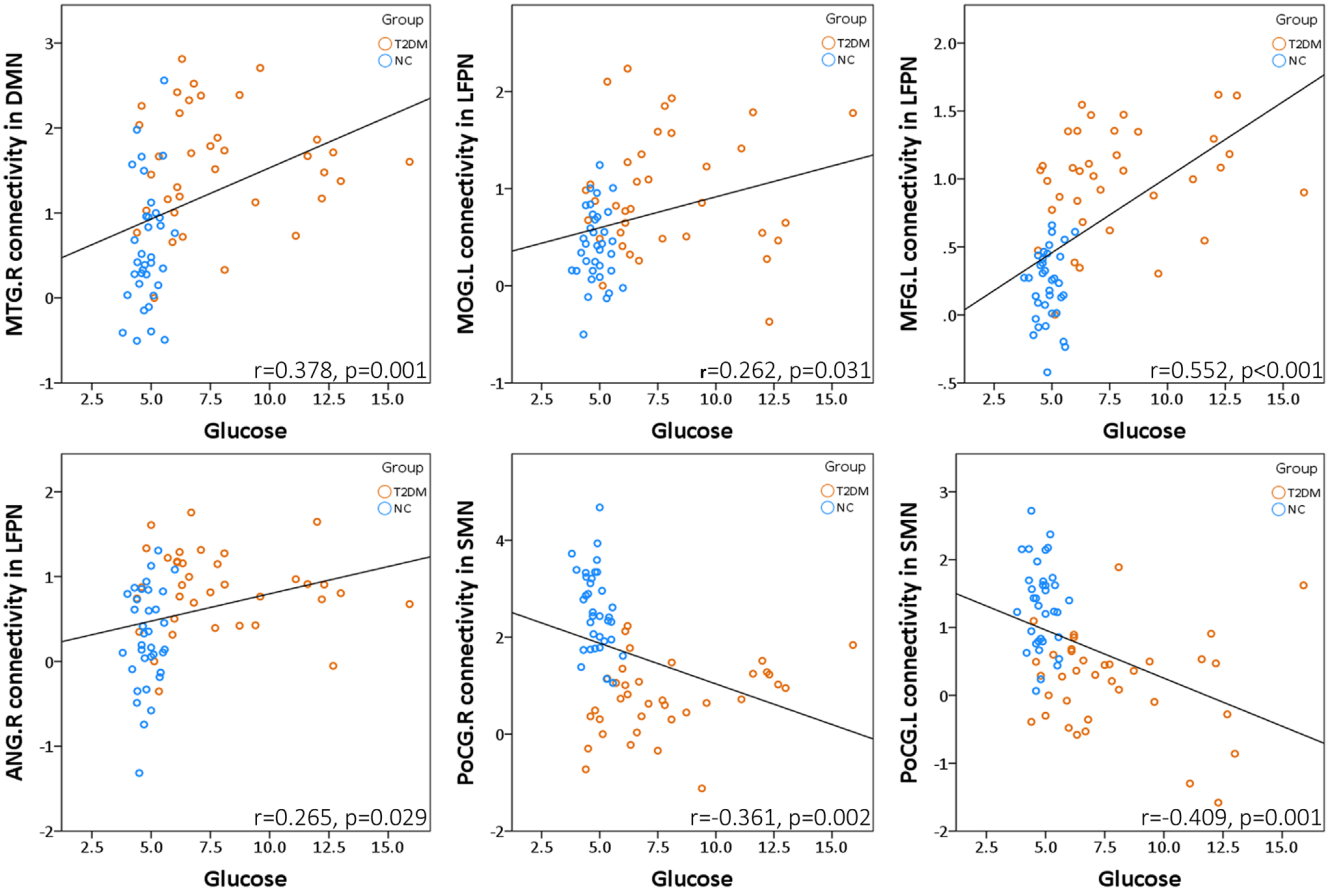
**Fasting plasma glucose levels were associated with abnormal functional connectivity in DMN, LFPN, and SMN**.

We assessed whether the observed connectivity changes in brain networks were associated with FPG levels. Pearson correlation analyses indicated that the higher glucose levels was associated with higher MTG·R connectivity in DMN (*r* = 0.378, *p* = 0.001), and connectivity in LFPN (MOG·L: *r* = 0.262, *p* = 0.031; MFG·L: *r* = 0.552, *p* < 0.001; ANG·R: *r* = 0.265, *p* = 0.029) in all participants after controlling for the effects of age, gender, and education. In addition, the glucose levels showed a negative correlation with the PoCG·R (*r* = −0.361, *p* = 0.002) and PoCG·L (*r* = −0.409, *p* = 0.001) connectivity in SMN (Figure 3).

## Discussion

In the present study, our results primarily showed that patients with T2DM exhibited deficits in several cognitive domains, including working memory, processing speed, and executive function. In particular, it could be speculated that some cognitive impairment was caused by the injury of the related brain network (Dai et al., 2014). In our subsequent analysis of the resting state functional networks, except for the DMN conforming to our anticipation, the connectivity within the LFPN and SMN was well altered in T2DM patients. More importantly, the FPG levels were correlated with abnormal functional connectivity.

In the neuropsychological tests, the scores of working memory, processing speed, and executive function in T2DM patients were significantly lower than those in healthy controls. In a further FPG-cognition correlation analysis, we found that higher FPG levels were associated with poorer working memory. Evidence from separate lines of research support our finding that diabetes had negative effects on cognition (Van Den Berg et al., 2010). Although a previous systematic review showed that abnormal glucose tolerance was associated with cognitive impairments, the mechanisms have yet to be clearly identified (Lamport et al., 2009). Therefore, we attempted to explain the suggested relationship between T2DM and impaired cognitive performance from a neuroimaging perspective.

A growing body of research has demonstrated that disconnection within and between networks is responsible for impairment in corresponding cognitive domains (Whitwell et al., 2011; Shu et al., 2012; Wang et al., 2013a; Chen et al., 2014a,c; Dai et al., 2014). In our study, we found that the disconnection phenomenon is specific to a subset of brain networks in non-demented T2DM patients, whereas other networks remain unaffected. By applying ICA to patients with T2DM, we found that the MTG·R of the DMN as well as the MOG·L, MFG·L, and ANG·R of the LFPN exhibited increased functional connectivity in T2DM patients. Similar results have been validated in aging processes, and increased functional connectivity in several regions, including the medial prefrontal and middle temporal cortices of the DMN was associated with higher beta-amyloid (Aβ), a dominant AD-associated pathological change, reflecting either a compensatory response or aberrant activity (Mormino et al., 2011). The areas of changed functional connectivity within the DMN and the LFPN found in our study also seem to be involved in early changes of AD. AD patients show increased functional connectivity in the MFG, which possibly compensates for the loss-of-function in the posterior cingulate and MTG (Cha et al., 2013). Coincidently, we found some support in a previous study that the connectivity between the posterior part of the DMN and inferior parietal lobule was increased in AD patients, potentially indicating a compensatory mechanism (Wang et al., 2013b). However, the compensatory theory is still ambiguous. Although controversial, this mechanism could account for the increased risk of brain dysfunction (Filippini et al., 2009). Taken together, one may suggest that the brain of non-demented T2DM patients produces a positive response, like increasing functional connectivity, to make up for cognitive declines, such as executive function and memory. Nevertheless, previous studies using an ROI-based approach have shown decreased connectivity between the posterior cingulate and other extensive areas (Zhou et al., 2010; Musen et al., 2012; Chen et al., 2014d; Hoogenboom et al., 2014). This difference could be due to the varying degree of disease in the patients. The T2DM patients in our study were recruited from the local community and had mild cognitive deficits, thus, may be at the disease’s early stage and compensate for brain injury by increasing network connectivity. Unfortunately, this mechanism will finally collapse due to injury accumulation and development of the disease. To the best of our knowledge, only a few studies have examined resting state functional connectivity in patients with T1DM, and one study found reduced connectivity in the MTG and temporal pole, and lateral occipital cortex and superior parietal lobule only in T1DM patients with microangiopathy (Van Duinkerken et al., 2012). However, different pathogenesis of T1DM and T2DM and discrepancies of age and disease duration could account for the variant results. On the other hand, the connectivity was diminished in the bilateral PoCG within the SMN in T2DM patients compared with controls, probably induced by loss of neurons and synaptic connections, which was somewhat consistent with functional and structural changes in the brain in healthy aging and diseases (Schulpis et al., 2001). Several consistent lines of evidence indicate that the SMN shows reduced resting state connectivity (Roski et al., 2013) and atrophied gray matter volume (Hafkemeijer et al., 2014) during aging. Additionally, long-standing T1DM patients showed reduced cortical thickness of the PoCG (Frokjaer et al., 2013). In summary, our results suggest that T2DM has a selective effect on the brain networks and corresponds well with changes that have previously been described in aging, AD, and T1DM.

Nevertheless, we did not find any differences in functional connectivity of the RFPN between T2DM patients and healthy controls. Research shows that age is strongly related to lateralization in multiple regions within the SMN, attention network, and frontal network (Agcaoglu et al., 2014). Additionally, a recent analysis revealed that extensive cerebral gray and white matter injury in subjects with T2DM is located predominantly in the left hemisphere (Yau et al., 2014). Similarly, some neurodegenerative disease and dementia patients exhibit injury primarily in the left rather than the right hemisphere, and hypometabolism is more susceptible to neurodegeneration in the left hemisphere (Loewenstein et al., 1989). This provides a possible explanation as to why the LFPN instead of the RFPN is disrupted in response to diabetes.

Furthermore, we assessed whether the observed connectivity changes in brain networks or FPG levels were associated with measures of cognition. The FPG levels showed a negative correlation with the backward digit span (*r* = −0.47, *p* = 0.006), ROCF-copy (*r* = −0.33, *p* = 0.062), and digit span (*r* = −0.31, *p* = 0.085) in the T2DM group (Figure S1 in Supplementary Material). Additionally, our results showed that the functional connectivity in the ANG·R within the LFPN was positively associated with ROCF-copy performance in non-demented T2DM patients after controlling for the effects of age, gender, and education (Figure S1 in Supplementary Material). This result suggested that the diabetic patients strived to remain at a higher cognitive level through a compensation mechanism that was used to strengthen network connectivity. A recent study found that the increased connectivity of the angular gyrus with the ventral attention network was related to higher executive function (Reineberg et al., 2015), which was basically identical to our result. The ROCF-copy test assesses the cognitive domain of visuo-spatial processing speed. Previous studies in T2DM patients revealed that the poorer transmission efficiency of the white matter network (Reijmer et al., 2013) and the reduced task-dependent activation (Chen et al., 2014b) were associated with a slowing of processing speed. These results indicated that declines in cognitive abilities closely correlate with functional or structural changes in the brain. Meanwhile, clinical indexes, such as insulin resistance, have shown a negative relationship with functional connectivity in the precuneus and inferior frontal gyrus (Musen et al., 2012), posterior cingulate, and MTG (Chen et al., 2014d). These studies suggested that decreased functional connectivity was associated with more serious injury caused by the disease. Overall, the altered resting state functional connectivity pattern in the present study may help to deepen our understanding of the brain injury mechanisms underlying cognitive deficits related to diabetes. A limitation to consider, however, is that we did not find any relationship between altered functional connectivity and cognitive performance in healthy controls. This may be due to the narrow but normal ranges of cognitive scores and resting state functional connectivity in the current control group. In other words, the reduced sensitivity of cognitive function variations to connectivity changes led to the non-significant results.

This study had several limitations. First, previous studies have reported the changed functional connectivity in the MTG (Xia et al., 2015) and the posterior part of DMN (Cui et al., 2015) of T2DM patients was associated with insulin resistance, which was not take into account in our study. Considering that using exogenous insulin could affect fasting plasma insulin level, we did not involve insulin resistance or C-peptide levels in the analysis. Second, it would be important to monitor the disease progression in our non-demented T2DM patients in order to investigate longitudinal changes between those who do and do not progress to AD. Therefore, both areas should be studied in future work.

## Conclusion

In summary, our current study suggests that T2DM selectively damages some resting state functional networks, including the DMN, LFPN, and SMN. To the best of our knowledge, we are the first group to study the brain mechanisms of cognitive deficits associated with T2DM from the perspective of functional networks in Chinese non-demented elderly. Our findings could help to understand the neural circuits of cognitive impairments and contribute to determine neuroimaging biomarkers for use in the early diagnosis and intervention of neurodegenerative diseases. Further studies with longitudinal data and a larger sample size are needed to verify our results and assess the values of these neuroimaging biomarkers.

## Author Contributions

Study concept and design: ZZ. Acquisition of data: YC, JZ, LL, SZ, and XL. Drafting of the manuscript: YC and ZL. Critical revision of the manuscript for important intellectual content: ZZ. Statistical analysis: YC, JZ, GT, and KC. Obtained funding: Dr. ZZ.

## Conflict of Interest Statement

The authors declare that the research was conducted in the absence of any commercial or financial relationships that could be construed as a potential conflict of interest. Despite having collaborated on research projects, the Associate Editor Jesus Avila, and the Review Editor Alberto Rábano, declare that the review has been carried out objectively.

## Footnotes

^1^http://rfmri.org/DPARSF

^2^http://mialab.mrn.org/software/gift/

## Supplementary Material

Figure S1 **Higher FPG levels were associated with worse cognitive function. Additionally, increased ANG·R functional connectivity within LFPN was associated with better cognitive performance in T2DM patients**.

## Abbreviations

AD, Alzheimer’s disease; ANCOVA, analysis of covariance; ANG·R, right angular gyrus; AVLT, Auditory Verbal Learning Test; Aβ, beta-amyloid; BABRI, Beijing Aging Brain Rejuvenation Initiative; BMI, body mass index; BNT, Boston Naming Test; BOLD, blood oxygen level dependent; CDT, Clock-Drawing Test; CVFT, Category Verbal Fluency Test; DMN, default mode network; ECN, executive control networks; fMRI, functional magnetic resonance imaging; FPG, fasting plasma glucose; FPN, frontal–parietal network; HbA_1C_, glycosylated hemoglobin; HDL, high density lipoprotein; ICA, independent component analyses; IRB, institutional review board; LDL, low density lipoprotein; LFPN, left frontal–parietal network; MFG·L, left middle frontal gyrus; MMSE, Mini-Mental-Status Examination-Chinese version; MOG·L, left middle occipital gyrus; MRI, magnetic resonance imaging; MTG·R, right middle temporal gyrus; MVN, medial visual network; PoCG, postcentral gyrus; RFPN, right frontal–parietal network; ROCF, Rey–Osterrieth Complex Figure test; ROI, region-of-interest; RSNs, resting state networks; SCWT, Stroop Color and Word Test; SDMT, Symbol Digit Modalities Test; SMN, sensorimotor network; SN, salience network; T2DM, type 2 diabetes mellitus; TC, total cholesterol; TG, triglyceride; TMT, Trail-Making Test.
