# Supplementary figures and images for: Selectively Disrupted Functional Connectivity Networks in Type 2 Diabetes Mellitus
Source: Front Aging Neurosci. 2015 Dec 11;7:233. doi: 10.3389/fnagi.2015.00233 (PMC4675853; doi:10.3389/fnagi.2015.00233)

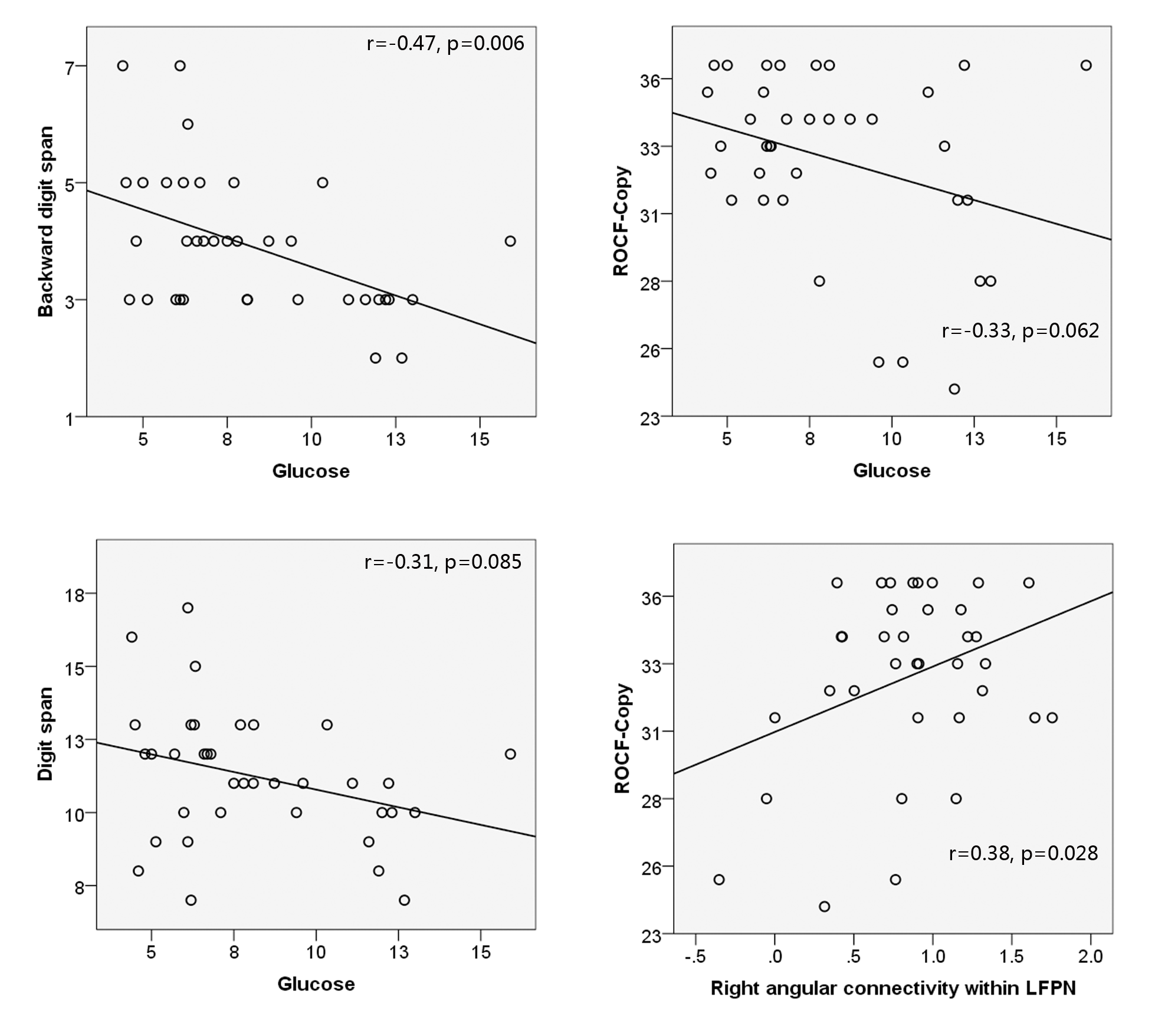

Supplement: Figure S1 — Higher FPG levels were associated with worse cognitive function. Additionally, increased ANG·R functional connectivity within LFPN was associated with better cognitive performance in T2DM patients. [file Image_1.TIF]
